# An elusive cause of recurrent hypoglycemia: delayed diagnosis of insulinoma treated with curative distal pancreatectomy in a resource-limited setting

**DOI:** 10.1093/omcr/omag188

**Published:** 2026-10-04

**Authors:** Yitagesu Aberra Shibiru, Shale Ali Kemal, Abenizer Berhanu Tulu, Dawit Dagne Moges

**Affiliations:** Adama Hospital Medical College, Adama, Ethiopia; Adama Hospital Medical College, Adama, Ethiopia; Adama Hospital Medical College, Adama, Ethiopia; Department of Radiology, Kidus Petros Specialized Hospital, Addis Ababa, Ethiopia

**Keywords:** Insulinoma, neuroendocrine tumor, Hypoglycemia, pancreatic neoplasm, distal Pancreatectomy

## Abstract

A 43-year-old woman presented with recurrent hypoglycemia and episodes of loss of consciousness. On admission, her blood glucose was 37 mg/dL (reference range: 70–99 mg/dl), with recovery after intravenous dextrose, fulfilling Whipple’s triad. She had a two-year history of dizziness, fatigue, diaphoresis, and neuroglycopenic symptoms, often relieved by frequent soft drink intake, leading to weight gain. She was previously misdiagnosed with a neurologic disorder, resulting in delayed evaluation. Laboratory findings showed elevated serum insulin levels of 65 μU/mL (reference range: 2.6–24.9 μU/mL) during hypoglycemia. Contrast-enhanced CT identified a 1.7 × 1.3 cm mass in the pancreatic body without metastasis. The patient underwent distal pancreatectomy with splenectomy. Postoperatively, hypoglycemic episodes resolved completely. Histopathology confirmed insulinoma. This case highlights the importance of considering insulinoma in patients with recurrent hypoglycemia. Delayed diagnosis may occur due to nonspecific symptoms and misdiagnosis. Careful clinical assessment, basic biochemical testing, and imaging can facilitate timely diagnosis and effective surgical management in resource-limited settings.

## Introduction

Insulinoma is an uncommon pancreatic neuroendocrine tumor characterized by endogenous hyperinsulinemic hypoglycemia, with an estimated incidence of approximately 4 cases per million population [1]. Although it is the most frequent functioning pancreatic neuroendocrine tumor, its overall occurrence remains low. Patients often present with nonspecific symptoms related to hypoglycemia, which can delay recognition and diagnosis, particularly in resource-limited settings [2].

## Case summary

We report the case of a 43-year-old woman who presented to our hospital with a history of loss of consciousness for 2 h. She was referred from a nearby private hospital after being found unresponsive at home. According to her family, she developed fatigue and dizziness prior to losing consciousness. On arrival, a random blood sugar measurement was 37 mg/dL. She was immediately treated with an intravenous bolus of 40% dextrose, followed by maintenance infusion of 10% dextrose. She gradually regained consciousness, although she remained disoriented, uncooperative, and not fully aware of her surroundings.

Her family reported that she had experienced similar episodes at home on multiple occasions, which usually improved after intake of soft drinks. They noted that on some days, she consumed up to 4–5 bottles of soft drinks to relieve her symptoms.

On further inquiry, the patient’s symptoms had begun approximately two years earlier, with recurrent episodes of lightheadedness, dizziness, fatigue, diaphoresis, irritability, and lack of interest in activities. These episodes were associated with documented low blood glucose levels, but she did not undergo further evaluation or follow-up. Over time, the episodes increased in frequency and severity, leading to recurrent loss of consciousness. Because of this, she eventually left her job and returned to live with her family.

Her functional status progressively declined; she became socially withdrawn, stopped communicating with her family, and required assistance with activities of daily living such as eating and dressing. To alleviate her symptoms, she frequently resorted to drinking large amounts of soft drinks, reportedly consuming up to 20 bottles per day, which resulted in significant weight gain.

Prior to her current presentation, she had visited multiple hospitals for evaluation of her recurrent loss of consciousness. At one facility, the episodes were considered to be possibly due to epilepsy, and she underwent brain MRI, which was unremarkable. Despite this, her symptoms persisted and the frequency of unconscious episodes increased. There was no history of medication use, exogenous insulin use or family history of similar symptoms.

On physical examination, the patient appeared well-looking and well-nourished, weighing 68 kg with a body mass index (BMI) of 26.5 kg/m^2^. Her vital signs at presentation were within normal limits: blood pressure 120/80 mmHg, pulse rate 84 beats per minute, respiratory rate 20 breaths per minute, and temperature 36.4°C. No other remarkable findings were noted on systemic examination.

The patient was admitted for observation, and a supervised 72-h fasting test was initiated. However, the test had to be discontinued due to recurrent episodes of severe hypoglycemia, with blood glucose levels dropping to as low as 22 mg/dL. Hypoglycemia persisted despite administration of maintenance intravenous fluids containing 10% dextrose, requiring multiple boluses of 40% dextrose and frequent bedside blood glucose monitoring.

### Laboratory

Routine laboratory investigations, including hematological, biochemical, and urinalysis parameters, were within normal limits. Given the strong clinical suspicion of insulinoma, additional endocrine evaluation was performed. The patient’s serum insulin level was elevated at **65 μU/mL** (reference range: 2.6–24.9 μU/mL). Due to resource limitations, supportive laboratory investigations, including C-peptide, proinsulin, β-hydroxybutyrate, and a sulfonylurea/meglitinide screen, could not be performed.

### Imaging

A contrast-enhanced abdominal CT scan revealed there was precontrast isodense and arterial phase marked homogenous enhancing pancreatic body mass measuring 1.7 cm ×1.3 cm. There was no evidence of invasion into the surrounding structures and no lymphadenopathy (Fig. 1).

**Figure 1:**
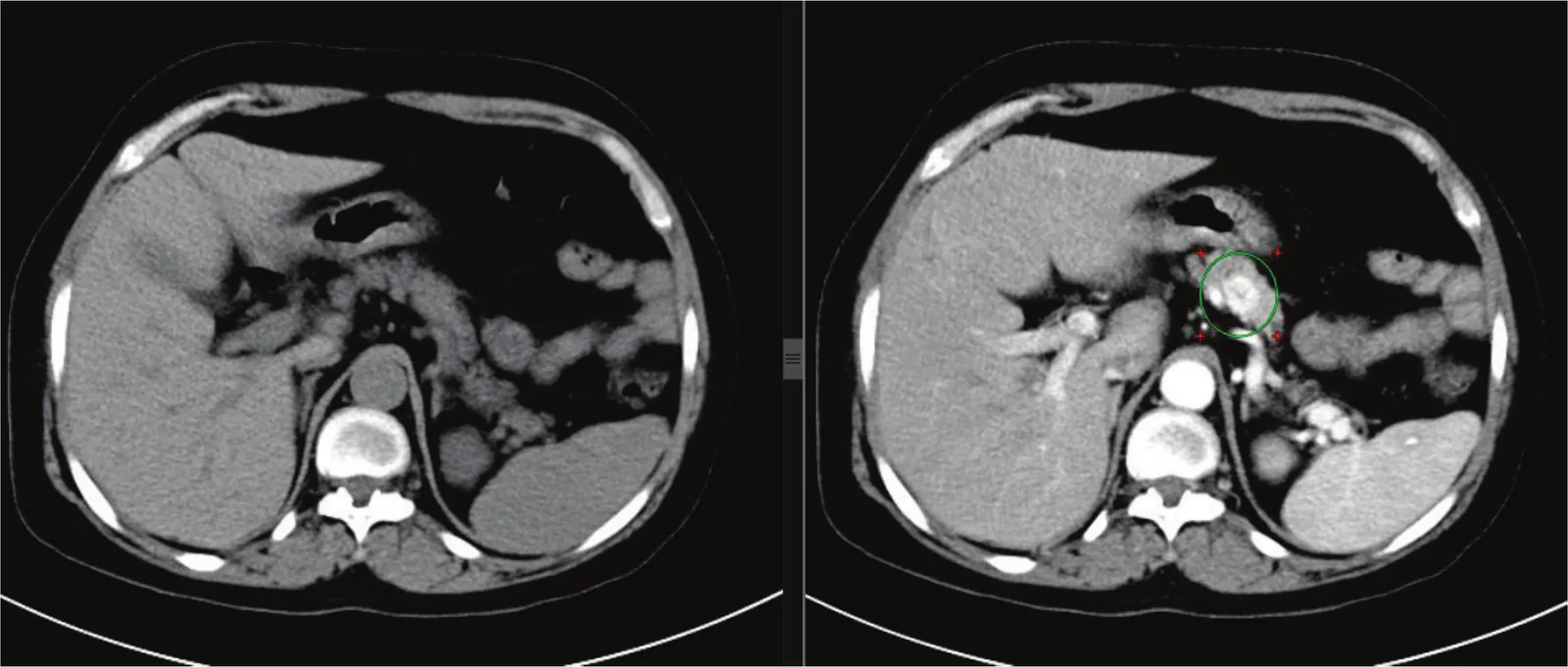
Abdominal CT with contrast axial image (A) show non contrast image mass at the body of the pancreas (B) contrast enhanced image of the mass homogenously enhanced mass measuring 1.7 by 1.3 cm at the body of the pancreases.

### Management

After counseling the family, the patient was prepared for surgery and taken to the operating theater. Through a bilateral subcostal incision, exploration revealed a firm mass in the body of the pancreas, with no evidence of lymphadenopathy. The tumor measured more than 2 cm and was located in close proximity to the main pancreatic duct. Although the clinical and biochemical findings strongly suggested a functional pancreatic neuroendocrine tumor (insulinoma), its oncologic behavior could not be determined preoperatively. Therefore, radical resection was considered the most appropriate management. After confirming negative surgical margins, a distal pancreatectomy with splenectomy was performed (Fig. 2). The distal pancreatic duct was identified and ligated with Prolene, and the pancreatic stump was reinforced with omentum. The total operative time was approximately three hours. The resected specimen was sent for histopathological examination.

**Figure 2:**
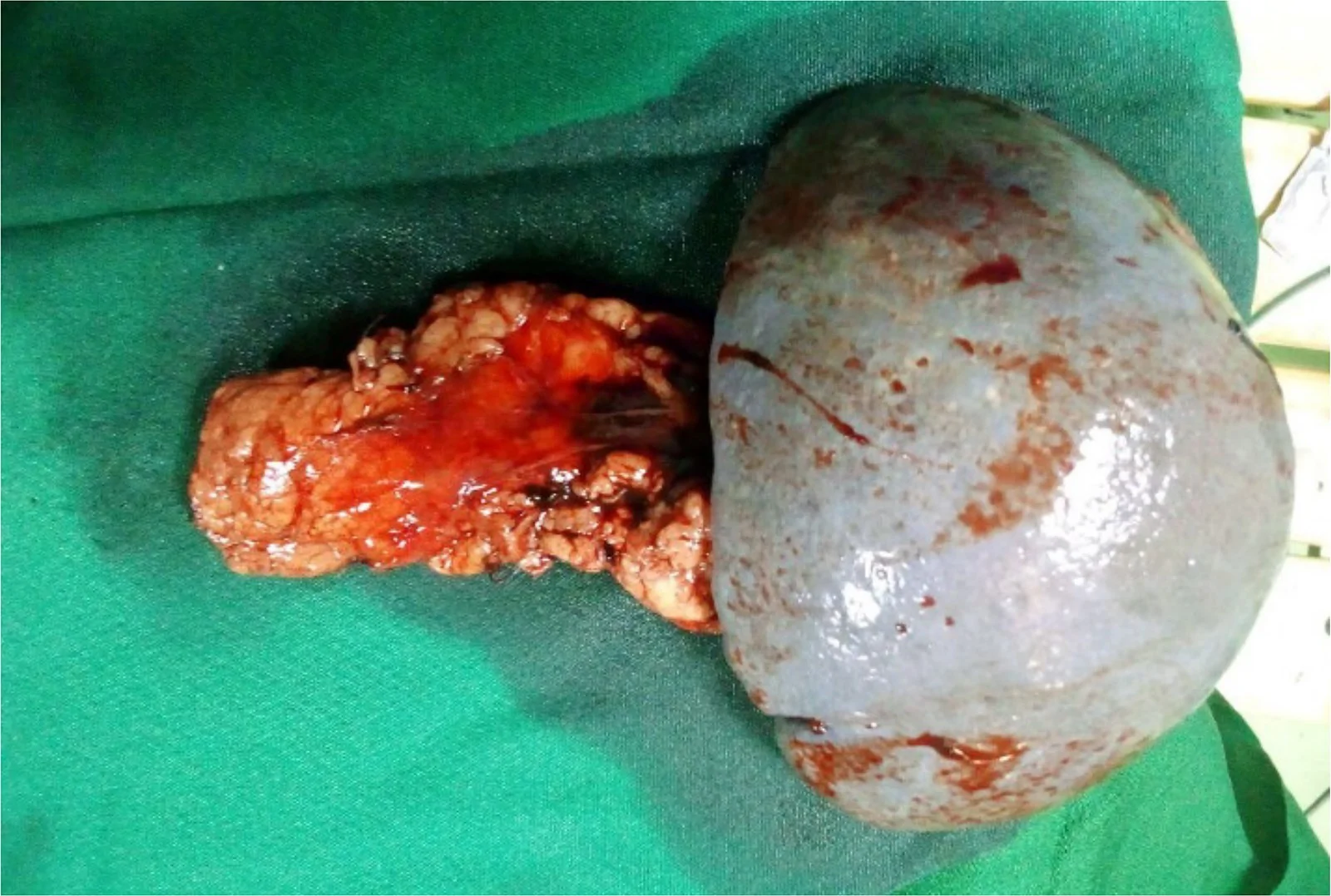
Excised distal pancreas with a mass on the left end and spleen. The mass indicated by white arrow.

Intraoperatively, about 20 min after skin incision, the patient developed hypoglycemia with a blood glucose level of 40 mg/dL, which was promptly corrected. She was subsequently maintained on a continuous infusion of 5% dextrose throughout the procedure. A surgical drain was placed before closure (Fig. 3).

**Figure 3:**
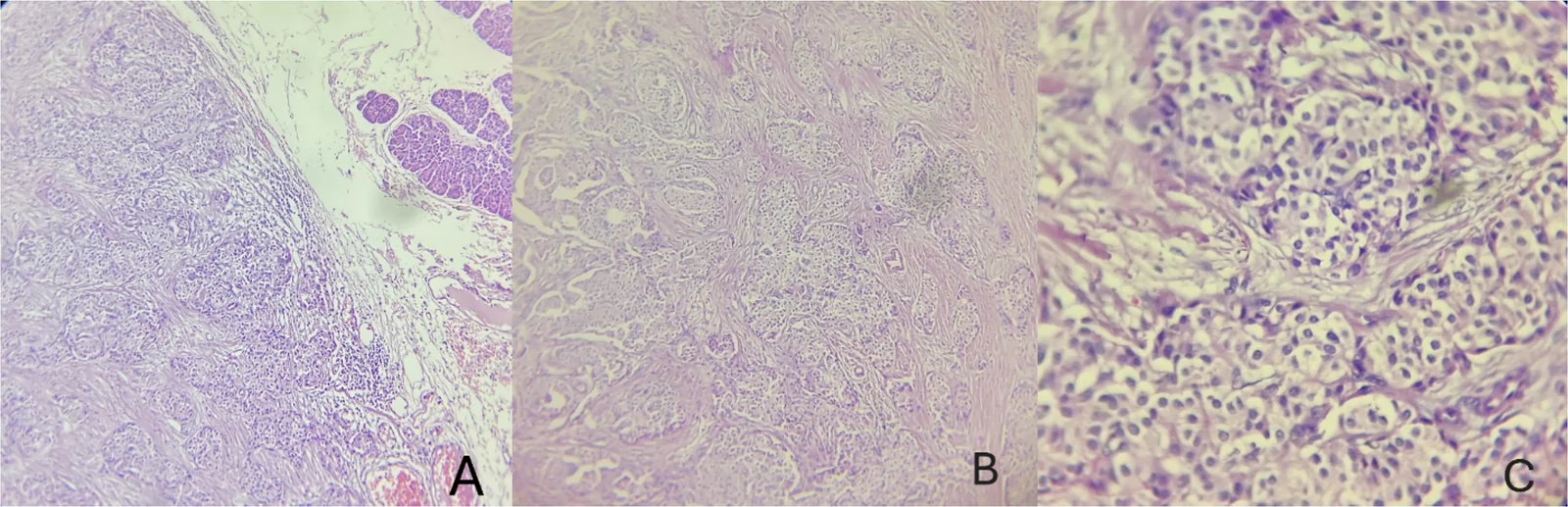
Histopathology image of the excised tissue (A) Low power view (40×) showing invasive nests of with adjacent unremarkable pancreatic parenchyma. (B) Medium power view (100×). (C) High power view (400×) showing nested pattern of monotonous cells demonstrating round nuclei with salt and pepper-like chromatin and abundant cytoplasm.

The patient was transferred to the surgical ward in stable condition. No further episodes of hypoglycemia occurred postoperatively. The drain was removed on the fifth postoperative day after having no output beyond the 3^rd^ day. The patient’s postoperative recovery was uneventful, and she was discharged in good condition on the 10th postoperative day. On subsequent post-operative follow-up her blood glucose and serum insulin remained normal.

## Discussion

The clinical manifestations of insulinoma are largely attributable to hypoglycemia and are typically categorized as autonomic (neurogenic) and neuroglycopenic symptoms [3]. In our patient, recurrent episodes of dizziness, fatigue, diaphoresis, and progressive neuroglycopenic features, including loss of consciousness, were evident over a two-year period. These features, combined with documented hypoglycemia and symptom resolution following glucose administration, fulfilled Whipple’s triad. Despite this, the patient experienced a prolonged diagnostic delay and was initially managed as having a neurologic disorder, a pattern reported in up to 20% of cases [4]. This diagnostic overlap arises because neuroglycopenic manifestations—such as confusion, altered behavior, seizures, and loss of consciousness—closely mimic primary neurologic conditions, including seizure disorders, transient ischemic attacks, and psychiatric illness, particularly when episodes are intermittent and not temporally linked to fasting or meals by the treating clinician. As a result, patients are often subjected to extensive neurologic work-up, including electroencephalography and neuroimaging, before hypoglycemia is considered as the underlying etiology.

An additional notable feature in this case was the patient’s adaptive behavior of frequent soft drink consumption to relieve symptoms, which led to significant weight gain. Compensatory hyperphagia and weight gain have been described in 20–50% of patients with insulinoma [5]. In this case, such behavior likely contributed to masking symptoms and delaying definitive evaluation, further reinforcing the misattribution of symptoms to a primary neurologic rather than metabolic cause.

Biochemical confirmation of insulinoma relies on demonstrating inappropriately elevated insulin levels in the presence of hypoglycemia. A supervised 72-h fasting test is considered the standard diagnostic approach and confirms the diagnosis in the majority of cases [6]. In our patient, the test was terminated early due to recurrent severe hypoglycemia; however, markedly elevated serum insulin levels during hypoglycemia supported the diagnosis.

Accurate preoperative localization is essential for surgical planning. In this patient, contrast-enhanced CT imaging identified a small, well-defined lesion in the pancreatic body without evidence of local invasion or metastasis. While endoscopic ultrasound has a reported sensitivity of approximately 82–85% [7], and selective arterial calcium stimulation with hepatic venous sampling may reach sensitivities of up to 93% in difficult cases [4], these modalities are often unavailable in low-resource settings. This underscores the importance of optimizing the use of accessible imaging techniques.

Surgical resection remains the definitive treatment for insulinoma and is associated with high cure rates ranging from 77% to 100% [8]. The choice of procedure depends on tumor size, location, and its relationship to the pancreatic duct. In this case, a distal pancreatectomy with splenectomy was performed due to the location of the tumor within the pancreatic body. The patient had an uneventful postoperative course, with complete resolution of hypoglycemic episodes, consistent with successful tumor removal.

This case highlights several important considerations. Insulinoma should be included in the differential diagnosis of recurrent hypoglycemia, particularly in patients presenting with neuroglycopenic symptoms. Misdiagnosis as a neurologic or psychiatric condition can significantly delay appropriate management [4]. Even in resource-limited settings, a combination of careful clinical assessment, basic biochemical evaluation, and available imaging can lead to accurate diagnosis and effective surgical treatment.

## Conclusion

Insulinoma should be suspected in patients presenting with recurrent hypoglycemia and Whipple’s triad, even in low-resource settings where advanced diagnostic tools are limited. Careful clinical evaluation, basic laboratory investigations, and accessible imaging modalities can still facilitate timely diagnosis. Surgical excision remains feasible and offers excellent outcomes, even in resource-constrained environments. To the best of our knowledge, this is the first reported case of insulinoma from Ethiopia, underscoring the need for greater awareness, early recognition, and reporting of such rare yet treatable conditions in low-resource countries.
